# Supplementary material for: The feasibility of the virtually delivered dementia lifestyle intervention for getting healthy together (DELIGHT) program for people living with dementia and their family/friend care partners
Source: PLoS One. 2025 Aug 12;20(8):e0328901. doi: 10.1371/journal.pone.0328901 (PMC12342274; doi:10.1371/journal.pone.0328901)
Supplement: S1 File — (DOCX) [file pone.0328901.s001.docx]

S1. Emergency Procedures

***Ensure that participant intake information and emergency contacts are accessible at the beginning of every class**

**If an incident occurs:**

1. Ask if the participant is okay.

- If non-responsive, meeting host will place all other participants into a separate breakout room and go to step 2a.
- If participant responds, request that they join a breakout room.
- If they are unable to reach their computer, host will place all other participants into a separate breakout room Go to step 2
- The secondary leader can continue with the class once a private room has been established.

1. Ask questions to determine if 911 is required:

- Is the participant able to respond?
- Is the participant able to move?
- Are there signs of cardiac emergency?
- Are there signs of glycemic emergency?
- Are there signs of stroke (F.A.S.T.)?

1. If 911 is required:

- Call 911
- Provide participant’s address from the intake form
- Follow directions from 911
- Stay online until EMS arrives
- Contact the participants emergency contact and let them know that EMS has been called

1. If 911 is NOT required

- Ask if participant is able/would like to continue class: If so, place participant back into main Zoom room.
- Encourage participant to follow up with health care provider if appropriate.
- Let participant know that you will be following up with them.

1. Follow up with participant and/or emergency contact.

**If a participant drops off the call:**

1. Have a volunteer phone them
   - If participant does not pick up, wait 2 minutes then call again
   - If participant picks up, ask them what happened and try to trouble shoot with them to reconnect when possible
   - If the participant is not able to reconnect
     1. Talk them through a cool down until they feel they are at resting level
     2. Inform them that the study leader will do a follow up call to try to further troubleshoot prior to next session
2. If the participant continues to not pick up
   - Phone emergency contact and inform them of the situation
3. If an emergency is suspected
   - phone 911
   - follow directions from 911

**If participant goes off screen or leaves the camera view:**

1. Verbally call out to see if the participant is still around
2. If they do not answer/come back into view, have volunteer call them

- If they answer
  - Ask them why they left and if everything is alright
  - Ask them if they would like to continue with the class
  - Remind them that if they are going off screen to give a thumbs up or a wave to let the study leader know they are safe
- If they do not answer
  - Wait 2 minutes and call again, then follow steps above
  - Phone emergency contact and inform them of the situation

1. If an emergency is suspected
   - phone 911
   - follow directions from 911

**If connection is lost by the leader:**

- The secondary leader who assists in delivery will take over the class if the study leader loses connection or the call is dropped.
- This individual will explain that the study leader lost connection due to some technical issues and he/she will join the class as soon as the issue has been resolved.

1. If this occurs during the exercise portion:

- The secondary leader will continue by keeping the participants active by very light intensity aerobic activities such as marching on spot to avoid any sudden exercise cessation.
- If the study leader is not successful in getting connected back in 10 minutes, then the secondary leader will announce that the class will no longer be continued due to the study leader’s ongoing technical issues and will end the class with a few cool down stretches.

1. If this occurs during the educational discussion portion:

- The secondary leader will continue by keeping the conversation going and utilizing the discussion prompts if necessary.
- The session will continue in this manner until the study leader returns or until the time of the program has ended.
