# Supplementary material for: The feasibility of the virtually delivered dementia lifestyle intervention for getting healthy together (DELIGHT) program for people living with dementia and their family/friend care partners
Source: PLoS One. 2025 Aug 12;20(8):e0328901. doi: 10.1371/journal.pone.0328901 (PMC12342274; doi:10.1371/journal.pone.0328901)
Supplement: S2 File — (DOCX) [file pone.0328901.s002.docx]

S2. Example 8-Exercise Circuit

1. Squat with resistance band​

2. Bent over row

3. Side leg raise​

4. Lateral and front arm raise

5. Single arm bicep curl

6. Bent over extension

7. Toe raise

8. Knee raises in recline position​

Cardio – 2-3 minutes

**Description of Exercises:**

| 1. **Squat with resistance band** |
| --- |
| **1.** Stand with feet shoulder-width apart and toes angled slightly out. To make it more challenging, stand on a band, ensure the tubing is spread evenly, hold the ends of the band at chest level.  **2.** Perform squatting action by hinging at the hips, pushing hips back, and bending the knees, keeping chest high and spine neutral (no rounding back).  **3.** Ensure your knees track in line with feet (no collapsing in or bowing out) and keep your weight on the midfoot. |

| 1. **Bent over row** | | |
| --- | --- | --- |
|  | **Level 1** | **Level 2** |
| **1.** Sit on the edge of a chair with one leg extended out in front and the band wrapped around your foot OR stand with your feet shoulder-width apart with the band anchored under both feet, knees bent, and hinged forward at the hips.  **2.** With arms fully extended, begin the movement by bending at the elbow, and pulling the band towards your ribs, keeping the shoulder blades squeezed together. Keep the elbows close to your side and do not let your arms wing out. Ensure wrists stay neutral throughout the movement.  **3.** Slowly return your arms to their starting position, not letting the tension of the band pull your arms back. | | |

| 1. **Side leg raise** | |
| --- | --- |
|  |  |
| **1.** Sit on the edge of a chair with one leg extended out in front with your heel on the floor OR stand up tall besides a sturdy chair.  **2.** With your hips facing forward, bring one leg out to the side.  **3.** Return your leg to its starting position in a controlled manner and repeat all repetitions with one leg. Make sure your core is engaged the entire time. Keep your torso straight and do not lean to either side or forward.  **4.** Repeat with the other foot on the ball once you completed all repetitions on this side. | |
|  |  |

| 1. **Lateral and front arm raise** |
| --- |
| **1.** Sit on the edge of a chair OR stand with feet hip-width apart, keeping a soft bend in the knee. Anchor a band under one foot and hold the other end in your hand, palm facing back.  **2.** Keeping your arms straight, slowly lift one arm up in front of you to your shoulder level or as far as you can without pain.  **3.** Slowly lower the arm down to the starting position.  **4.** Slowly lift one arm to the side to your shoulder level as far as you can without pain, then repeat. |

| 1. **Single arm bicep curl** | | |
| --- | --- | --- |
|  |  |  |
| **1.** Sit on the edge of a chair OR stand with a band anchored under both feet.  **2.** Hold both ends of the band with palms facing forward and arms straight down.  **3.** Hinge at elbow, one at a time to curl hands up to shoulders while keeping upper arm close to the torso, elbows tucked in and wrists in a neutral position. Ensure core is engaged to prevent any torso movement.  **4.** Lower your hand under control to straighten arms while keeping chest high and shoulders in a neutral position. | | |

| 1. **Bent over extension** |
| --- |
| **1.** Stand up tall in front of a sturdy chair. Hinge forward at the hips and place hands on the back of chair, maintaining a neutral spine.  **2.** While stabilizing hips, extend one leg directly behind you, keeping it straight. Do not rotate hips. Maintain a slight bend in the standing leg.  **3.** Return slowly to the starting position and repeat all repetitions with one leg.  **4.** Once reps are completed, shift weight to the other leg and complete a full set on the other side.   \| 1. **Toe raise** \| \| --- \| \| **1.** Sit on the edge of a chair OR stand upright with feet shoulder-width apart. Hold onto a chair for support if needed.  **2.** Raise the toes off the floor and go onto your heels without bending the knees.  **3.** Lower the toes under control to return to the starting position and repeat. \| |

| **8. Knee raises in recline position** |
| --- |
| **1.** Sit on the edge of a chair and lean back slightly without touching the back of the chair.  **2.** Lift one knee up as far as your range of motion allows, be sure to engage your core.  **3.** Return slowly to starting position then repeat on the other leg. That will count as 1 rep. Continue alternating legs for target repetitions. |
| **Cardio** |

Cardio can include any movement that is repetitive and increases your heart rate, for example: marching
