# Supplementary material for: The feasibility of the virtually delivered dementia lifestyle intervention for getting healthy together (DELIGHT) program for people living with dementia and their family/friend care partners
Source: PLoS One. 2025 Aug 12;20(8):e0328901. doi: 10.1371/journal.pone.0328901 (PMC12342274; doi:10.1371/journal.pone.0328901)
Supplement: Attachment — Submitted filename: DELIGHT_Response_to_Reviewers #3.docx [file pone.0328901.s003.docx]

**Response to Reviewers**

Thank you for asking us to address the outstanding issues related to data availability.

**1. Thank you for your response. We understand that you would now like to update your data availability statement to read as follows: "The anonymized quantitative data will now be available through the Figshare open access registry. However, the qualitative data contains sufficiently identifying information and the University of Waterloo Research Ethics Board has not given us approval to share the interview transcripts. The qualitative data will be made available upon request to the University of Waterloo Research Ethics Board."**

The anonymized quantitative data will be available through the Figshare open access registry and the specific link will be provided upon acceptance of the manuscript. However, the qualitative data contains sufficiently identifying information and the University of Waterloo Research Ethics Board has not given us approval to share the interview transcripts. The qualitative data will be made available upon request to the University of Waterloo Research Ethics Board. The contact email is researchoffice@uwaterloo.ca
